# Associations of Conflict-Related Trauma and Ongoing Stressors with the Mental Health and Functioning of West Papuan Refugees in Port Moresby, Papua New Guinea (PNG)

**DOI:** 10.1371/journal.pone.0125178

**Published:** 2015-04-29

**Authors:** Alvin Kuowei Tay, Susan Rees, Jack Chen, Moses Kareth, Sylvester Lahe, Russell Kitau, Kura David, Joyce Sonoling, Derrick Silove

**Affiliations:** 1 Psychiatry Research and Teaching Unit, Liverpool Hospital, School of Psychiatry, University of New South Wales, Sydney, Australia; 2 South Western Sydney Clinical School, Liverpool Hospital, Faculty of Medicine, University of New South Wales, Sydney, Australia; 3 Simpson Centre for Health Services Research, Faculty of Medicine, University of New South Wales, Sydney, Australia; 4 Division of Public Health, School of Medicine and Health Sciences, University of Papua New Guinea (UPNG), Port Moresby, Papua New Guinea; 5 Port Moresby General Hospital and School of Medicine and Health Sciences, University of Papua New Guinea (UPNG), Port Moresby, Papua New Guinea; Harvard Medical School, UNITED STATES

## Abstract

Documentation is limited in relation to the mental health of the people of West Papua, a territory that has been exposed to decades-long political persecution. We examined associations of traumatic events (TEs) and current stressors with mental disorder and functioning, amongst 230 West Papuan refugees residing in six settlements in Port Morseby, Papua New Guinea (PNG). We used culturally adapted modules to assess exposure to TEs and mental disorders. Current stressors and functioning were assessed using modifications of measures developed by the World Health Organization (WHO). 129 of 230 respondents (56%) reported exposure to at least one traumatic event (TE), including: political upheaval (36.5%), witnessing or hearing about family members tortured and murdered (33.9%), and not being able to access medical care for family members (33%). One fifth of respondents (47, 20.4%) experienced exposure to high levels of TEs (16 to 23). 211 (91.7%) endorsed at least one or more ongoing stressors, including: exposure to illicit substance use in the community (91.7%), problems with safety and the protection of women (89.6%), no access to legal rights and citizenship (88.3%), and lack of adequate shelter and facilities (85.2%). A quarter (26.9%) met criteria for one or more current mental disorder, and 69.1% reported functional impairment ranging from mild to extreme. Mental disorder was associated with being male (adjusted odds ratio=2.00; 95% CI=1.01-3.97), and exposure to the highest category of ongoing stressors (AOR=2.89; 95% CI=1.08-7.72). The TE count showed a dose-response pattern in its relationship with functional impairment, the greatest risk (AOR=11.47; 95% CI=2.11-62.37) being for those experiencing the highest level of TE exposure (16-23 events). West Papuans living in settlements in Port Moresby reported a range of TEs, ongoing stressors and associated mental disorders characteristic of populations exposed to mass conflict and persecution, prolonged displacement, and ongoing conditions of extreme hardship.

## Introduction

Repeated concerns have been raised concerning the impact of prolonged conflict and associated violation of human rights on the general and mental health of the indigenous people of West Papua [1, 2]. However, lack of access to the territory by the media, human rights groups, and health researchers has limited systematic documentation of these issues. With the exception of a small study undertaken in Australia [3], there are no data concerning the mental health of refugees from West Papua. We assessed the mental health and functioning of a displaced population of West Papuans living in conditions of prolonged statelessness in Port Moresby, the capital of Papua New Guinea.

There is little awareness internationally of the history of mass conflict in West Papua. The territory occupies the western half of the New Guinea landmass, lying to the north of Australia. West Papua was transferred from Dutch colonial rule to Indonesia in 1963 after a period of conflict concerning the future sovereignty of the territory. Indonesia subsequently annexed West Papua based on the results of a referendum that is widely regarded as flawed in that the vote was not representative of the indigenous people as a whole [4].

A low-grade armed conflict has ensued in West Papua in which the Indonesian military has attempted to suppress the pro-independence movement. During this prolonged period of conflict (1969 to the present), there have been repeated allegations made by reputable human rights organizations of abuses being perpetrated by the Indonesian military against the indigenous people, including instances of torture, rape, arbitrary detention and mass displacement of populations [4].

The ongoing state of persecution and conflict has led to a substantial exodus of refugees into neighbouring Papua New Guinea (PNG), and further afield, including Australia and Europe, with estimates suggesting that that over 10,000 West Papuans have fled their homeland in successive waves over the course of the past 50 years [5, 6]. The majority of refugees in PNG occupy makeshift camps on the border, locations that are dangerous and difficult to access [7, 8]. However, a number of displaced persons, particularly those who arrived in PNG in the 1980s, have relocated to “settlements” (or shanty towns) in Port Moresby where they live under conditions of extreme poverty and deprivation with limited legal rights or recognition of their refugee status. Refugees in the settlements around Port Moresby face challenges in many aspects of their lives, including social marginalization, insecure residency, threats to their physical safety in a city with one of highest rates of violence in the world, limited educational opportunities, unemployment, and exposure to the multiple stressors of prolonged displacement [9, 10].

A focus on West Papuan refugees is timely given increased international attention to the ongoing conflict in that territory. In 2013, mass protests were staged in the province to mark the 52^nd^ anniversary of the brief period of West Papuan independence following Dutch colonial rule [11]. In 2014, the European Parliament Subcommittee on Human Rights commissioned an inquiry into mounting allegations of human rights abuses in the province [12]. At the same time, a Citizens’ Tribunal conducted in Australia revealed multiple incidents of torture and murder during an alleged massacre by Indonesian Special Armed Forces on the Papuan island of Biak in 1988 [13]. In addition, the UN High Commissioner for Human Rights has publicly condemned the widespread violence used to suppress political dissidents and the curtailment of freedom of expression and association in West Papua [14].

However, major restrictions on entry to the province have presented ongoing obstacles to the systematic documentation of the health and mental health of West Papuans residing in the province. A focus on refugees who have fled the territory therefore offers an opportunity to reveal both the extent of human rights violations experienced by West Papuans and the consequences of these experiences on the community’s mental health and functioning. In addition, the study offers the opportunity to test some key issues of general importance regarding the long-term impact of past exposure to traumatic events (TEs) and post-migration stresses on the mental health and functioning of a refugee group. An ongoing controversy in the field relates to the importance of past TEs as opposed to post-migration stressors in determining the ongoing mental health of refugees [15]. Some critics in the field have claimed that past research and interventions have given excessive attention to the long-term psychological consequences of TEs in contrast to the effects of immediate stressors and deprivations that refugees commonly experience in the post-migration environment [16]. Resolving the relative roles of TEs and ongoing stresses to mental health outcomes is important in striking the correct balance when designing interventions for refugees, particularly in relation to the extent to which the focus should be on the psychological consequences of past TE exposure or on socio-economic and other assistance programs aimed at alleviating conditions of poverty and other hardships in the immediate, post-migration environment.

In addressing these issues, it is important to define outcomes more precisely, in particular, by distinguishing between mental disorder and psychosocial functioning, constructs that are inter-related but not identical. For example, survivors of conflict may experience subjective symptoms of mental disorder but, with some effort, manage to function adequately. Conversely, it is possible that exposure to the multiple TEs of persecution may result in the undermining of the individual’s capacity to function without leading to frank mental disorder.

In summary, building on a preliminary study undertaken in Australia [17], we report the findings of a cross-sectional, community-based study in order to provide much needed data concerning the mental health and functioning of West Papuan refugees, in this instance, the community residing in Port Moresby. Our aims are to identify: 1. the range and prevalence of TEs that the population has experienced; 2. the types and severity of ongoing stressors associated with their current living conditions; 3. the overall prevalence of current common mental disorders; and 4. associations of socio-demographic factors, past TEs and ongoing stressors with current mental disorder and psychosocial functioning.

## Materials and Methods

### Design

As of January, 2014, the United Nations High Commissioner for Refugees (UNHCR) estimated that approximately 90% of the 9378 refugees living in Papua New Guinea originated from West Papua [6]. Our focus on the Port Moresby based segment of the West Papuan population in PNG was determined by several factors, including the frequent movement of refugees residing near the border, inaccessibility to these areas by road or other forms of transport, and risk to the safety of researchers in travelling to these localities. Prior to our study, we had established contact with, and received a strong expression of willingness by the Port Moresby West Papuan population to document the history of conflict, ongoing conditions of life and mental health needs of the community living in the settlements surrounding the city.

We conducted a cross-sectional survey across the six settlements in Port Moresby where West Papuan refugees are known to be concentrated. Standard approaches to epidemiological sampling (such as using a door-to-door survey of a population of known size and location) was not feasible given the absence of census data identifying West Papuans, and the dispersion of this minority group across a larger population of PNG nationals who also reside in the settlements. Hence we adopted a key informant, targeted sampling approach, which involving two phases [18]. First, we conducted an extensive process of location mapping and qualitative data gathering during several field visits (October–December 2010), working in close collaboration with key leaders of the West Papuan community. Selection of the survey sites was based on the triangulation of information provided by community leaders, government officials, international organizations, local university staff, and United Nationals High Commissioner for Refugees. Drawing on all these sources, we were able to make a firm estimate that 90% of West Papuan refugees in Port Moresby lived in the six identified settlements, geographically defined communities characterized by high density, makeshift housing, and few facilities. These settlements are named Hohola, Rainbow, Six-Mile, Eight Mile, Nine-Mile, and Tokarara/Waigani. Again, based on triangulation of all available information, our conservative estimate was that there were 250 adult West Papuans including men and women, residing in these settlements at the time of the survey.

Phase two involved research personnel (a West Papuan refugee from Australia working with counterparts trusted by the community) mapping the location of the adult West Papuan refugee population within these settlements, based again on information already gathered, and supplemented by door-to-door inquiries covering the entire population of these locations. Eligible respondents were male and female West Papuan refugees who were 16 years or older at the time of the survey, including those born in West Papua and those of West Papuan descent (defined as at least one parent being Papuan) born in PNG. Twenty of the estimated 250 eligible respondents had dispersed to other areas of Port Moresby or further afield and could not be contacted, yielding a response rate from the identified pool of 92%.

Our prior consultations with the community indicated that older members of the community originating from West Papua are proficient in Bahasa Indonesian (the lingua franca in the homeland) as well as Tok Pisin (the pigeon English widely spoken throughout PNG). Given that members of the community originated from many parts of West Papua, subgroups also spoke one or more of the numerous indigenous or “mother” tongue languages, which differ substantially across regions, preventing the use of any one language for general communication amongst the refugee group as a whole. Members of the younger generation are proficient in both English and Tok Pisin but not always in Bahasa Indonesian.

### Ethics statement

Two consultations open to all members of the community were organized during which the research team met with community leaders and refugee residents who were informed about all aspects of the survey. Ethical permission for the study was provided by the University of New South Wales Human Research Ethics Committee and the Medical Research Council of PNG Ethics Committee. The study was conducted according to the ethical provisions of the Declaration of Helsinki for medical research involving human subjects.

The majority of respondents gave written consent prior to the commencement of the interviews wherever possible. Witnessed oral consent was obtained in some cases where respondents were illiterate. Interviews were conducted in a private location or within the home of the respondent, depending on their preference.

### Field personnel training

West Papuan refugees employed as research assistants were trained to conduct the interviews. We selected field interviewers on the basis of their fluency in Bahasa Indonesian, English, and Tok Pisin, the three relevant languages, their positions of trust in the community, and their competence and commitment to the project. Interviews participated in a three-week training program conducted by the research team, a clinical psychologist and a bilingual West Papuan researcher from Australia. The training sessions focused on sensitizing field workers to mental health problems relating to conflict-affected populations; developing interviewing skills and role-play; ensuring familiarity with the research protocol; and practising the administration of the assessment battery. Interrater reliability was assessed by the psychologist and a PNG medical practitioner trainee in Psychiatry who independently re-interviewed five study participants who had been assessed by the respective field worker. There was a high level of interrater agreement in assigning individual diagnoses between field workers and professional personnel (interrater agreement = 93.1%, k = 0.86, z = 8.65, p<0.001).

In addition, two field workers received additional training on data management including data checking and entry into an electronic database under the supervision of the psychologist. Field interviewers received a certificate upon completion of the training program. Weekly post-training supervision sessions were conducted with the field interviewers during the study period (January–May, 2013) either on-site or by telephone from Australia. All completed interviews were checked and entered into an electronic database by the field workers and further cross-checked for potential errors and inconsistencies. All interviews were stored in a locked filing cabinet in a secured location prior to being transported to Australia.

### Assessment modules

Four culturally adapted and tested interview modules were applied to assess: (1) exposure to Traumatic Events (TEs); (2) ongoing stressors; (3) relevant DSM-IV and DSM-5 (Diagnostic and Statistical Manual for Mental Disorders) [19, 20]; and (4) physical wellbeing. In addition, a modified version of the WHO-DAS was applied to assess psychosocial functioning [21].

#### Exposure to Traumatic Events (TEs)

We assessed exposure to TEs according to a list of 23 items (experienced and/or witnessed), the content being based on a review of the historical and contemporary literature in the field adapted to the specific context following a process of extensive consultation with the West Papuan refugee community. Each TE was dated according to key historical periods relating to the conflict and period of migration (spanning the period 1960 through to 2010). We included both conflict-related and civilian types of TEs (such as intimate partner violence, vehicle accidents, assaults, natural disasters). The measure demonstrated sound reliability based on the Kuder-Richardson reliability coefficient (KR20 = 0.94). To indicate the prevalence of exposure, we generated a summary index for five types of TEs broadly clustered according to their common characteristics: war-related, family deaths and separations, witnessing torture and abuse, childhood-related adversities, and lack of access to medical care in times of emergency. Following the convention in the field, we applied a composite index of TE exposure (or summary trauma count) representing the addition of endorsed items in our analysis, consistent with the large body of studies in the post-conflict mental health field [22]. To facilitate consistent reporting and interpretation of odds ratios in our multiple logistic regression models, we followed convention by dividing the TE count into four ascending ordinal groupings based on the distribution of the overall trauma count (0, 1–3, 4–15, 16–23) [23, 24]. The lowest grouping was used as the reference point for all comparisons.

#### Ongoing stressors

We assessed ongoing stressors using an adapted version of the Humanitarian Emergency Settings Perceived Needs (HESPER) scale developed by the World Health Organization (WHO) in collaboration with the Institute of Psychiatry, King’s College, London [25]. The measure comprises 26 items, each rated as a “serious problem” or “not a serious problem“. Assessments of the HESPER in community studies undertaken in Iraq, Jordan, and Nepal produced sound evidence of interrater-reliability (ICC = 0.99 in Jordan, 0.99 in Nepal; k = 0.66–1.0 across sites), test-retest reliability (ICC = 0.96 in Jordan, 0.73 = Nepal; k = 0.07–1.0 across sites), and internal consistency (α = 0.8 in Jordan, α = 0.8 in Nepal). In addition, the HESPER yielded a high level of convergence with the WHO Quality-of-Life (WHOQOL-100) with a significant correlation between the total number of endorsed stressors and the total WHOQOL score (r = -0.629 in Jordan, r = -0.469 in Nepal).

We adapted the measure to the local culture and context via an extensive series of consultations in which all items were evaluated in both focus groups and individual informant interviews undertaken with members of the West Papuan refugee community. The objective was to ensure that each item was clearly understood and endorsed as relevant to the community. Item wording was modified slightly and some items added during the process. The adapted item pool yielded a high level of reliability, producing a KR20 of 0.93. Given that the stressor items were inter-correlated, we generated a composite stress index or count (an addition of the endorsed items, each assigned a score of 1). The stress items received a high level of endorsement (only 2.6% reported no stressors whereas over 95% reported at least one). Consistent with our approach to analysing TEs, we derived ascending ordinal groupings based on the distribution of the stress count score (0–15, 16–20, >21). Given the high overall level of exposure, the lowest group (the reference group in analyses) included those who reported 0 to 15 forms of stressors.

#### Mental disorders and psychological reactions

We used a purpose-designed mental disorder module that could readily be applied by lay interviewers in the field to assess nine common mental disorders and psychological reactions (assessed for the previous 12 month period) including both DSM-IV and DSM-5 definitions. The mental disorders were chosen based on the existing literature and their practical application in the contemporary refugee field [22, 26–33]. The disorders/reactions included: posttraumatic stress disorder (PTSD); major depressive disorder; generalized anxiety disorder; panic disorder; somatic symptom disorder; persistent complex bereavement related disorder (a newly proposed DSM-5 diagnosis); separation anxiety disorder, psychosis; and intermittent explosive disorder.

Development of the module commenced with the establishment of items derived from both editions of the DSM for each category. Adaptation involved a process of iterative consultation with individual psychiatrists in PNG who are from a broadly similar Melanesian background as West Papuans. All psychiatrists endorsed the constructs underlying each diagnosis, the constituent symptoms, and the applicability of all categories within the local context, thereby offering some evidence of the face validity of the module. Extensive consultations (focus groups, informant interviews) then were undertaken with members of the West Papuan community to review the comprehensibility, relevance and cultural appropriateness of items in a community with minimal knowledge of, or contact with, western mental health constructs or services. Many of the symptoms or constellations of symptoms had broad equivalents in terms used in Bahasa Indonesia by West Papuans. For example, in relation to PTSD symptoms, terms commonly referred to were “*waspada*” (hypervigilance), “*menghindari*” (avoidance), “*kehilangan minat*” (loss of interest), “*dijaga*” (startle response), “*sakit hati*” (anger and resentment), and “*tidak percaya*” (loss of trust).

In applying the module, algorithms based on the DSM IV/5 diagnostic criteria were used to determine caseness for each disorder. We conducted a convergence study to test the diagnostic module against a gold standard clinical interview. Given the constrained number of persons receiving individual diagnoses, we divided participants into those who were cases of mental disorder (presence of one or more current disorders) and non-cases (no disorder). We compared the findings of the lay administered field interview with case assignments according to the Structured Clinical Interview for DSM-IV (SCID) (provisionally modified to allow both DSM-IV and DSM-5 diagnoses to be made), administered blind by an experienced Australian-based psychologist who speaks Bahasa Indonesian. There was a high level of convergence between the two assessments: AUC = 93%; CI = 0.87–0.98; sensitivity = 93%; specificity = 95%. In the present analysis, we report distinctions between cases and non-cases determined according to DSM-IV criteria.

#### Psychosocial functioning

Psychosocial functioning was assessed using the 12-item version of the WHO Disability Assessment Schedule (WHODAS 2.0) [21], based on the International Classification of Functioning and Health. Each item is rated on a 5-point Likert scale: none = 1; mild = 2; moderate = 3; severe = 4; extreme = 5. The WHODAS has been widely used across epidemiological surveys worldwide, yielding sound internal consistency (Cronbach *α* = 0.86). We derived a categorical index of functioning based on a summary score of all items (0 for scores of 0 or 1; 1 for those who scored 2 and above).

#### General health

Physical health was assessed by an item derived from the Australian Bureau of Statistics National Survey of Mental Health and Wellbeing (based on the MOS SF Scales) [34, 35] inquiring how the respondents felt about their physical health on a five-point Likert scale: excellent (1), very good (2), good (3), fair (4), or poor (5). A general health status index was generated (yes/no) using 5 as the cut-off point. A score of 5 indicates poor health (lower values indicate good-moderate health).

#### Transcultural assessment of the modules

The assessment battery was translated into Bahasa Indonesia from English by a professional translator and back-translated and checked for errors by a bilingual mental health clinician following standard transcultural instrument development procedures [36]. Poorly worded or ambiguous items were removed or rephrased to ensure comprehensibility. Transcultural assessment of the modules applied in the study was undertaken amongst members of the West Papuan community, focusing on four core indicators: content relevance, linguistic equivalence (correct interpretation of items), technical equivalence (appropriateness of response scale), and completeness (equivalence of construct [37]. Iterative adjustments were made to the modules to ensure that these criteria were met (details of qualitative results are available upon request). The final test was that latter focus groups endorsed the transcultural validity of the modules without suggesting any further modifications.

### Statistical analysis

Intraclass Correlation (ICC) values were calculated to assess for the possible effects of clustering by settlement and family unit. Due to the low ICCs yielded for both mental disorder and psychosocial outcome variables (settlement-level = 0.05; family-level = 0.00) no adjustments were made to control for design effects in further analyses. Multiple logistic regression models were applied to examine for associations between TEs and ongoing stressors, and each of the two outcomes, aggregated current mental disorder and psychosocial functioning, respectively. Socio-demographic variables were assessed in preliminary univariate analyses for possible inclusion as covariates in the multiple regression analysis. Bivariate analyses for socio-demographic variables that yielded statistical associations at the p<0.05 level were retained as covariates in the relevant multivariate analyses.

Log function tests and the Hosmer and Lemeshow method were used to assess for potential misspecification errors in the logistic models. Tests of associations are reported as odds ratios (ORs) in the univariate models and as adjusted odds ratios (AORs) with 95% confidence intervals (CIs) in the multivariate models. Statistical significance was assessed at p<0.05. STATA version 13 was used to analyze the data.

## Results

The sample included 230 West Papuan adults (men 137, 59.5%; women 93, 40.4%) whose mean age was 36.9 (sd = 15.44) years. Of these, almost half were born in West Papua (n = 107, 46.5%; mean age = 49.2 years, sd = 12.2), the remained being first generation descendants born in PNG (n = 123, 52.4%; mean age = 26.3 years, sd = 8.5). Table 1 presents the sociodemographic characteristics and mental health status of the sample and the subgroups born in West Papua and PNG.

**Table 1:** Sociodemographic Characteristics and Mental Health of Refugees Born In West Papua and PNG (N = 230).

		WP-born		PNG-born		Total participants	
Sociodemographic Characteristics and mental health outcomes		n	%	n	%	n	%
		107	100	123	100	230	100
**Sex**	Female	31	29	62	50.4	93	40.4
	Male	76	71	61	49.6	137	59.6
**Age (in years)**	16–23	1	0.9	55	44.7	56	24.4
	24–32	12	11.2	47	38.2	59	25.7
	33–50	35	32.7	19	15.5	54	23.5
	>51	59	55.1	2	1.6	61	26.5
	Mean (SD)	49.2	(12.2)	26.3	(8.5)	36.9	(15.4)
	Median	51.0		24.0		32.5	
**Marital status**	Single	29	27.1	65	52.9	94	40.9
	Married/engaged	78	72.9	58	47.2	136	59.1
**Highest level of education attained**	Primary	96	89.7	114	92.7	210	91.3
	Secondary	6	5.6	5	4	11	4.8
	Tertiary	5	4.7	4	3.3	9	3.9
**Occupation**	Unemployed	68	63.6	78	63.4	146	63.5
	Employed (fishing/farming, NGOs)	39	36.5	45	36.6	84	36.5
**Prevalence of TE domains** ^1^	War-related trauma	87	81.3	17	13.8	104	45.2
	Witnessing murders	84	78.5	24	19.5	108	47.0
	Traumatic losses	85	79.4	9	7.3	94	40.9
	Access to emergency medical care	74	69.2	6	4.9	80	34.8
	Childhood related adversities	32	29.9	7	5.7	39	17.0
**Exposure to TEs** ^2^	0	13	12.2	88	71.5	101	43.9
	1–3	7	6.5	27	22.0	34	14.8
	4–15	40	37.4	8	6.5	48	20.9
	16–23	47	43.9	0	0	47	20.4
**Exposure to ongoing stressors**	0–15	9	8.41	38	31.0	47	20.4
	16–20	10	9.4	16	13.0	26	11.3
	21–25	88	82.2	69	56.1	157	68.3
**Current mental disorder** ^3^	No disorder	73	68.2	95	77.2	168	73.0
	1 disorder	21	19.6	15	12.2	36	15.7
	2 or more disorder	8	7.5	9	7.3	17	7.4
	3 or more disorder	5	4.7	4	3.3	9	3.9
**Functional impairment** ^4^	WHODAS total score below 13	14	13.1	57	46.3	71	30.9
	WHODAS total score above 13	93	86.9	66	53.6	159	69.1
	WHODAS mean score (SD)	24.9	(8.7)	17.2	(7.8)	20.7	(9.0)
**General health**	Reported excellent-moderate health	56	52.3	90	73.2	146	63.5
	Reported poor health	51	47.7	33	26.8	84	36.5

^1^ We grouped TEs into five broad clusters according to their common characteristics: war-related experiences, traumatic losses, witnessing torture and abuse, childhood-related adversities, and lack of access to emergency medical care.

^2^ Categorization of TEs and stressors was based on ascending ordinal groupings of exposure of approximately equal distributions of TEs and stressors.

^3^ Current mental disorders defined based on DSM-4/5 criteria (0 = non-cases, 1 = case) assessed in the past 12 month period include posttraumatic stress disorder; major depression; generalized anxiety disorder; panic disorder; somatic symptom disorder; persistent complex bereavement related disorder; separation anxiety disorder, psychosis; and intermittent explosive disorder.

^4^ A cutoff of 13 derived from total score distribution.

Participants born in West Papua had lived in PNG for a mean of 27 years (sd = 10.28). There were more males (χ^2^_1_ = 10.92, P<0.001) and older persons (t(228) = 16.65, P<0.001) in that subpopulation than those born in PNG. Refugees had arrived in PNG in four distinct waves extending from the 1960s through to 1990s:1960–1970 (11%); 1971–1980 (23%); 1981–1990 (57%); 1991–2013 (9.8%). Half of participants resided in two settlements: Hohola (65, 28.2%) and Rainbow (47, 20.4%).

Fifty-nine percent (n = 136) were married or engaged and the remainder were single, separated or widowed (n = 94, 40.9%). Ninety-one percent (n = 210) had completed primary education; small numbers had completed high school (n = 11, 4.8%) and higher education (university, vocational training) (n = 9, 3.9%). Over half of the whole sample was unemployed (n = 146, 63.5%) with the remainder being occupied in fishing, farming, government, and NGO sectors. The PNG-born had higher levels of employment. There were no statistically significant differences in other socio-demographic characteristics and prevalence of current mental disorder between those born in West Papua or PNG.

Table 2 shows the prevalence of exposure to TEs. The majority (n = 129, 56%) reported exposure to at least one TE, the order of frequency for TEs being: forced to live in poor conditions during conflict (86, 37.4%); exposure to political upheaval (84, 36.5%); witnessing or hearing about family members and/or strangers being tortured or murdered (78, 33.9%); not being able to access emergency medical care for family members (76, 33%); and traumatic losses involving deaths and disappearances of family members (74, 32.2%). Fourteen percent (34) reported exposure to 1 to 3 counts of TEs; 20.9% to 4–15; and 20.4% 16–23. Men reported higher level of exposure to TEs compared to female (χ^2^_3_ = 30.09, P<0.001).

**Table 2:** Prevalence of Exposure to Traumatic Events.

Conflict-related TEs	n (%)
Forced to live in poor conditions due to ongoing violence	86 (37.4)
Direct experience of war for political reasons	84 (36.5)
Home intentionally destroyed	80 (34.8)
Lack of shelter because of conflict	79 (34.3)
Humiliated in front of other people	73 (31.7)
Forced to go into hiding during war	70 (30.4)
Involved in active combat as freedom fighters	68 (29.6)
Held captive or imprisoned	54 (23.5)
Torture	35 (15.2)
Abducted by members of other political groups	25 (10.9)
Disappearances of family members	74 (32.2)
Separated from family members	71 (30.9)
Multiple deaths of family members	67 (29.1)
Forced to abandon family members during war	69 (30)
Not being able to perform cultural ceremonies for the dead	37 (16.1)
Witnessing strangers tortured	78 (33.9)
Hearing about family members tortured and murdered	78 (33.9)
Witnessing rape and sexual abuse	38 (16.5)
Witnessing dead bodies	60 (26.1)
Witnessing violence at home	31 (13.5)
Physical abuse during childhood	26 (11.3)
Not being able to access medical care for family members	76 (33)
Not being able to access medical care for self	68 (29.6)

Older West Papuans (mean age = 49 years) reported significantly greater exposure to TEs specifically related to war and conflict compared to younger members of the community (mean age = 26 years) (χ^2^_3_ = 134.33, P<0.001). Overall, the older West Papuans (mean age = 49.2 years, sd = 12.2) reported higher rates of exposure to all five clusters of TEs (war-related, witnessing murders, traumatic losses, access to emergency medical, and childhood adversities) compared to those born in PNG (mean age = 26.3 years, sd = 8.5). Table 3 shows rates of endorsement of five categories of TEs by age groups and country of origin.

**Table 3:** Prevalence of Traumatic Events by Age Groups and Country of Origin (West Papua/PNG).

	WP-born (n = 107)									
TEs	War-related		Witnessing murders		Traumatic losses		Access to emergency care		Childhood adversities	
Age (years)	N	col %	n	col %	n	col %	n	col %	n	col %
	87	100	84	100	85	100	74	100	32	100
**16–23**	0	0	1	1.2	0	0	0	0	0	0
**24–32**	7	8	5	6	7	8.2	4	5.4	1	3.2
**33–50**	28	32.2	28	33.3	26	30.6	23	31.2	14	43.8
**>51**	52	59.8	50	59.5	52	61.2	47	63.5	17	53.1
	**PNG-born (n = 123)**									
	**War-related**		**Witnessing murders**		**Traumatic losses**		**Access to emergency care**		**Childhood adversities**	
**Age (years)**	n	col %	n	col %	n	col %	n	col %	n	col %
	17	100	24	100	9	100	6	100	7	100
**16–23**	9	52.9	13	54.2	4	44.4	3	50	4	57.1
**24–32**	6	35.3	6	25	4	44.4	3	50	3	42.9
**33–50**	2	11.8	4	16.7	1	11.1	0	0	0	0
**>51**	0	0	1	4.2	0	0	0	0	0	0

Table 4 shows the prevalence of exposure to ongoing stressors. By far the majority (211, 91.7%) endorsed at least one listed item. The most highly endorsed items were: concerns about the use of illicit substances in the community (211, 91.7%); safety and protection for women from violence (206, 89.6%); lack of legal rights and citizenship (203, 88.3%); lack of proper shelter or housing (196, 85.2%); lack of access to basic facilities (196, 85.2%); general safety in the community (196, 85.2%); limited assistance from government organizations and international agencies (196, 85.2%); and prolonged displacement (190, 82.6%). One fifth (20.4%) of respondents had a stressor count ranging from 0 to 15; 11.3% a range of 16 to 20; and 68.3%, 21 to 26.

**Table 4:** Prevalence of Exposure to Ongoing Stressors.

Ongoing Stressors	n (%)
Use of alcohol and illicit substances in the community	211 (91.7)
Safety and protection for women from violence	206 (89.6)
Lack of legal rights	203 (88.3)
Lack of proper shelter and housing	196 (85.2)
Lack of access to a clean toilet	196 (85.2)
General safety in the community	196 (85.2)
Lack of aid and support	196 (85.2)
Lack of access to clean water	191 (83)
Lack of support for vulnerable populations	191 (83)
Being displaced from home	190 (82.6)
Lack of respect in the community	190 (82.6)
Livelihoods	190 (82.6)
Lack of information re aid and welfare	186 (80.1)
Difficulties moving between villages	184 (80)
Lack of food	182 (79.1)
Lack of support and care for family members	180 (33.9)
Lack of hygiene	180 (33.9)
Lack of clothes and blankets	175 (76.1)
Feeling very distressed	174 (75.7)
Limited educational opportunities	170 (73.9)
Separation from family members	168 (73)
Mental illness in the community	166 (72.1)
Lack of access to healthcare	154 (67)

Over a quarter (62, 26%) met criteria for one or more mental disorders (15.6% a single disorder, 11.3% comorbid disorders). The most common current mental disorders were (in order of prevalence): separation anxiety disorder (22, 10%); persistent complex bereavement disorder (DSM-5 disorder) (22, 10%); panic disorder (20, 9%); posttraumatic stress disorder (15, 7%); generalized anxiety disorder (14, 6%); intermittent explosive disorder (14, 6%); somatization disorder (10, 4%); major depression (8, 3%); and psychotic disorder (4, 2%). 69.1% reported functional impairment ranging from mild to extreme.

Men had a high prevalence of mental disorder (according to overall case rate) than women (χ^2^_1_ = 5.97, p = 0.02). Older age (χ^2^_3_ = 30.81, P<0.001) and country of birth (χ^2^_1_ = 29.66, P<0.001) were related to functional impairment. Specifically, the oldest age category had the greatest level of functional impairment (>50 years) compared to participants of other ages. There was little statistical association between mental disorder and functional impairment (χ^2^_1_ = 1.77, P = 0.183). In addition, older West Papuans reported poorer general health than younger members of the community born in PNG (χ^2^_1_ = 10.71, P<0.01).

### Current mental disorder

Tables 5 and 6 show the results of the two sets of univariate and multivariate logistic regression analyses examining associations of TEs and stressors with mental disorder and psychosocial functioning respectively.

**Table 5:** Association of Exposure to Traumatic Events, Ongoing Stressors, and Sociodemographic Characteristics with Current Mental Disorder amongst West Papuan Refugees (N = 230).

		Current mental disorder^1^	
		Univariate	Multivariate
Sociodemographic characteristics and mental health indices		Unadjusted Odds Ratio^2^ (OR) (95% CI)	Adjusted Odds Ratio (AOR) (95% CI)
**Country of origin**	Total		
	PNG-born	1 [Reference]	1 [Reference]
	WP-born	1.58 (0.88–2.84)	0.63 (0.23–1.72)
**Sex**	Female	1 [Reference]	1 [Reference]
	Male	2.19 (1.16–4.13)**	2.00 (1.01–3.97)*
**Age (in years)**	16–23	1 [Reference]	1 [Reference]
	24–32	1.03 (0.43–2.44)	0.65 (0.23–1.88)
	33–50	0.95 (0.39–2.30)	0.93 (0.31–2.79)
	>50	2.00 (0.89–4.49)	0.93 (0.31–2.79)
**Marital status**	Single	1 [Reference]	1 [Reference]
	Married/engaged	1.93 (0.98–3.39)	1.98 (0.80–3.92)
**Highest level of education attained**	Primary	1 [Reference]	1 [Reference]
	Secondary	1.44 (0.89–3.45)	1.41 (0.70–3.40)
	Tertiary	2.29 (0.78–2.34)	0.90 (0.34–2.39)
**Employment**	Unemployed	1 [Reference]	1 [Reference]
	Employed (fishing farming, NGOs)	1.03 (0.57–1.89)	1.00 (0.52–1.92)
**Exposure to TEs**	0	1 [Reference]	1 [Reference]
	1–3	0.55 (0.19–1.59)	0.59 (0.20–1.72)
	4–15	0.95 (0.42–2.15)	0.73 (0.31–1.70)
	16–23	2.82 (1.36–5.88)**	1.63 (0.70–3.77)
**Exposure to ongoing stressors**	0–15	1 [Reference]	1 [Reference]
	16–20	1.62 (0.44–5.96)	1.63 (0.44–6.05)
	21–25	3.29 (1.31–8.25)**	2.89 (1.08–7.72)*
**Functional impairment**	WHODAS total score below 13	1 [Reference]	1 [Reference]
	WHODAS total score above 13	1.57 (0.81–3.04)	1.33 (0.62–2.85)
**General health** ^3^	No	1 [Reference]	1 [Reference]
	Yes	1.50 (0.82–2.72)	1.21 (0.64–2.30)

^1^ Current mental disorders assigned as cases (met criteria for one or more mental disorders assessed in the past 12-month period) or non-cases (reference category) according to DSM-4/5 criteria for posttraumatic stress disorder; major depression; generalized anxiety disorder; panic disorder; somatic symptom disorder; persistent complex bereavement related disorder; separation anxiety disorder, psychosis; and intermittent explosive disorder.

^2^ Odds ratios and adjusted odds ratios are calculated with 95% confidence interval; multivariate logistic regression models included covariates: sex, country of origin, TE exposure, ongoing stressors (significant at p<0.05 in univariate models).

^3^ We applied 5 as a cutoff (poor) for general health.

*P<0.05;

**p<0.01

**Table 6:** Association of Exposure to Traumatic Events, Ongoing Stressors, and Sociodemographic Characteristics with Functional Impairment amongst West Papuan Refugees (n = 230).

		Functional impairment^1^	
		Univariate	Multivariate
Sociodemographic characteristics and mental health indices		Unadjusted Odds Ratio^2^ (OR) (95% CI)	Adjusted Odds Ratio (AOR) (95% CI)
**Country of origin**	Total		
	PNG-born	1 [Reference]	1 [Reference]
	WP-born	5.74 (2.95–11.15)**	1.50 (0.52–4.31)
**Sex**	Female	1 [Reference]	1 [Reference]
	Male	0.94 (0.53–1.67)	0.56 (0.28–1.09)
**Age (in years)**	16–23	1 [Reference]	1 [Reference]
	24–32	0.83 (0.40–1.73)	0.59 (0.26–1.34)
	33–50	2.82 (1.23–6.47)**	0.95 (0.32–2.77)
	>50	9.03 (3.14–25.96)**	1.60 (0.36–6.99)
**Marital status**	Single	1 [Reference]	1 [Reference]
	Married/engaged	2.11 (1.20–3.74)**	1.20 (0.60–2.40)
**Highest level of education attained**	Primary	1 [Reference]	1 [Reference]
	Secondary	0.87 (0.48–1.56)	0.91 (0.47–1.76)
	Tertiary	0.90 (0.40–1.99)	0.95 (0.39–2.32)
**Employment**	Unemployed	1 [Reference]	1 [Reference]
	Employed (fishing farming, NGOs)	0.83 (0.47–1.49)	0.89 (0.45–1.75)
**Exposure to TEs**	0	1 [Reference]	1 [Reference]
	1–3	3.59 (1.48–8.68)**	3.81 (1.53–9.46)**
	4–15	5.52 (2.35–12.96)**	2.95 (1.03–8.41)*
	16–23	24.84 (5.72–10.97)**	11.47 (2.11–62.37)**
**Exposure to ongoing stressors**	0–15	1 [Reference]	1 [Reference]
	16–20	0.91 (0.33–2.44)	0.82 (0.27–2.45)
	21–25	1.46 (0.73–2.90)	0.58 (0.26–1.28)
**Current mental disorder**	No disorder	1 [Reference]	1 [Reference]
	1 disorder	1.75 (0.75–4.09)	1.48 (0.57–3.83)
	2 or more disorder	1.20 (0.40–3.57)	0.93 (0.26–3.40)
	3 or more disorder	0.75 (0.12–4.62)	0.22 (0.02–2.09)
**General health** ^3^	No	1 [Reference]	1 [Reference]
	Yes	2.84 (1.49–5.49)**	1.93 (0.93–4.02)

^1^ A cutoff of 13 derived from mean WHODAS score distribution.

^2^ Odds ratios and adjusted odds ratios are calculated with 95% confidence interval; multivariate logistic regression models included covariates: sex, country of origin, TE exposure, ongoing stressors (significant at p<0.05 in univariate models).

^3^ We applied a cutoff score of 5 (poor) for general health.

*P<0.05;

**p<0.01

Univariate associations found that mental disorder cases differed from non-cases in relation to TEs and stressors. Specifically, more cases endorsed the highest category of TEs (OR = 2.82; 95% CI = 1.31–8.25) and the highest category of stressors (OR = 3.29; CI = 1.31–8.25). Multivariate analyses adjusting for relevant covariates indicated that mental disorder cases comprised more men (AOR = 2.00; CI = 1.01–3.97) and those with the highest category of exposure to ongoing stressors (AOR = 2.89; CI = 1.09–7.72).

### Ongoing functional impairment

Univariate analysis showed a dose-response relationship between TEs and functional impairment. Those endorsing the highest TE category (16–23 TEs; OR = 24.84; CI = 5.72–10.97) had the highest odds ratio for functional impairment, and conversely, those in the lowest TE exposed category had the smallest odds ratio (13 TEs: OR = 3.59; CI = 1.48–8.68) compared to the reference category who did not experience any TEs.

The dose-response association between TE categories and functional impairment was sustained in the multiple logistic regression analysis, with the AOR being particularly high for those with the greatest level of trauma exposure (AOR = 11.47; CI = 2.11–62.37).

## Discussion

Our study is the first to examine associations between the TEs of mass conflict and post-displacement stressors with the mental health and psychosocial functioning of West Papuans residing in Port Moresby, PNG. Our findings reveal the extent of the ongoing deprivations and hardships facing the displaced community living in settlements in Port Moresby. The older members of the community (mean age 49 years) represent a long-term displaced population, many being exposed to extreme human rights abuses and persecution. Older men had higher rates of mental disorder, general health problems and functional impairment, consistent with their direct exposure to warfare and human rights violations in the homeland. The findings offer much needed empirical support for allegations of widespread human rights violations being perpetrated against West Papuans in their home country over a prolonged period of time.

The level of functional impairment was relatively independent of the presence or absence of mental disorder, supporting our general impressions that members of the community, particularly older men born in West Papua, may experience difficulty in managing their daily lives but do not inevitably succumb to frank mental disorder. Importantly, the TE count showed a dose-response relationship with functional impairment, indicating the potential for traumatic experiences related to human rights violations to exert a lasting effect on the survivor’s capacity to cope, particularly in settings of displacement.

Prior to considering the inferences that can be drawn from our findings, we detail the strengths and limitations of our study. The study represents the first survey of its kind amongst West Papuan refugees displaced to PNG. Random epidemiological sampling was not possible given that this minority group is dispersed within a larger indigenous population from PNG. As a consequence, we had to rely on triangulation of information in our estimation of the numbers of West Papuan refugees and their location in settlements in Port Moresby. We achieved a high response rate based on a stepwise approach to identifying all eligible West Papuans residing in the identified settlements. The minority who were not identified had inter-married and/or dispersed into the wider PNG population.

We applied a comprehensive assessment protocol that had been tested for its cultural and contextual validity and psychometric properties within the same population. Nevertheless, the risk of transcultural measurement error in relation to our translated and adapted modules cannot be dismissed, particularly given the linguistic complexity of developing versions in the three most widely spoken languages (English, Bahasa Indonesian, Pidgin). Constraints in the sample size obliged us to aggregate individual mental disorders into a composite group of “cases”. This meant that we could not undertake fine-grained analysis of relationships of patterns of trauma and ongoing stressors with individual mental disorder categories.

Because of the overall high level of stressors in the community, we were obliged to include those with substantial levels of exposure (0–15) in defining our reference group in multivariate analyses. That constraint may have attenuated real differences between those with intermediate levels of stressors and the reference group, accounting for the observation that only those falling into the highest category of stressors had increased rates of mental disorder.

The population under study was heterogeneous with some members of the community born in West Papua and others in PNG. Although the former were more likely to experience conflict-related TEs, the younger group were at risk of exposure to high levels of violence in the settlements and Port Moresby in general. Diversity in the population did have the advantage of allowing identification of the older group of men born in West Papua and exposed to the most extreme human rights trauma, as those experiencing the greatest level of psychosocial dysfunction. Our extensive engagement with the community suggests that the older West Papuan-born population, particularly those who were exposed to extensive human rights violations during the conflict, have found it most difficult to adapt and function in the environment of the settlements in Port Moresby. In contrast, all members of the community are exposed to ongoing stressors and deprivations such as lack of adequate shelter and inadequate water and food.

In examining models of mental disorder and functioning respectively, we include socio-demographic factors, TEs and ongoing stressors, using multivariate analyses that allow identification of the unique contribution of each factor. However, our analysis could not take into consideration the impact of migration as a possible factor in its own right as a potential contributor to mental disorder, given that we did not have access to a comparison, non-emigrant group remaining in West Papua. It is not feasible at present to undertake studies in West Papua itself because of restrictions on researchers entering the country. Nevertheless, it may be possible to undertake future studies with larger samples of refugees residing in PNG to allow closer analysis of subgroup differences in relation to risk to mental disorder within this heterogeneous refugee group.

Finally, given that West Papuan refugees invariably are opponents of the Indonesian occupation, we could not dismiss the risk of bias in reporting levels of exposure to past human rights abuses. Nevertheless, the pattern of TEs reported is consistent with mounting evidence of widespread abuses occurring in West Papua produced by human rights organizations and other inquiries focusing on the nature of the conflict in West Papua [4, 12, 38]. As refugees, however, our study group is likely to have experienced a concentration of TEs and other adversities. While the experiences of our sample therefore may be regarded as broadly indicative of the nature and consequences of exposure to the typical traumas of conflict in the West Papuan homeland, the extent of these experiences within the small population studied cannot be regarded as representative of the experiences of the generality of West Papuans living in the province.

One-third of respondents reported experiencing multiple forms of human rights abuses in the homeland, the most common being the traumas related to political conflict, violent deaths and disappearances of family members, witnessing or hearing about family members/strangers tortured or murdered, and lack of access to medical care during times of emergency. Furthermore, the older members of the community reported the greater proportion of war-related TEs. The known history of the conflict adds face validity to these reports in that it is well established that politically motivated human rights violations in West Papua date back as far back as the 1960s, when a period of intensifying conflict occurred between pro-independence supporters and the Indonesian Special Forces [4]. A substantial number of West Papuans began fleeing their homeland in successive waves to seek asylum in the neighbouring PNG, with the largest group of refugees arriving in the 1980s [5]. Our data concerning the level of violence and persecution reported by refugees therefore are consistent with the available information from historical and medical sources [1, 4] and from our past studies undertaken with Papuan refugees resettled in Australia [3].

In our analyses, we included country of birth to adjust for potential qualitative differences in exposure and type of TEs experienced in the two environments (West Papua and PNG). A notable finding was that although the younger generation of West Papuans born in PNG had been spared direct exposure to conflict in the homeland, they were vulnerable to experiencing TEs related to violence in Port Moresby where exposure to rape and other sexual assault, communal conflict, and experiences of extreme difficulty in accessing emergency medical care are common.

By far the majority of refugees (91%) endorsed at least one form of ongoing stressors. In particular, refugees reported widespread concerns about the safety of themselves and their children in the community. The use of illicit substances and alcohol, and lack of enforcement of the rule of law were identified as major sources of stress, consistent with known conditions of endemic violence in Port Moresby in general [10]. Concerns about the impact of intimate partner violence on women in the community were evident, an area of ongoing public health concern given the lack of access to physical and psychological services for women survivors [39].

Many refugees had lived for up to four decades in Port Moresby since their arrival, and only a minority have achieved the status of naturalized citizens. As a consequence, the majority continue to live as “stateless” persons without access to the rights and protections of citizenship. Lack of recognition of their refugee status is further compounded by an intergenerational cycle of adversity characterized by extreme poverty and severe hardships, with the majority of families having limited access to medical services, adequate housing, and the capacity to acquire essential skills for employment (in an environment where unemployment in general is high). Accommodation is over-crowded, consisting mainly of shanty dwellings of poor quality, often housing multiple families with few facilities. Rights to land and property often is in dispute, governed by complex traditional law with citizens of PNG often claiming ownership [40]. In spite of their ethnic affinity with the host population (West Papuans and PNG nationals share a common Melanesian background), refugees still encounter discrimination and challenges in advancing professional careers for those with qualifications.

More than a quarter (27%) of respondents met criteria for one or more current mental disorders. These findings are consistent with studies amongst other post-conflict and refugee populations [22]. In addition, our data show that males had higher rates of current mental disorder, consistent with the greater exposure of men to TEs in West Papua. High levels of ongoing stressors were specifically associated with mental disorder in both univariate and multivariate analyses, even after controlling for the effects of past exposure to TEs. Although the highest category of exposure to TEs predicted mental disorders at the univariate level, the odds ratio became insignificant in multivariate analysis, possibly because of the attenuating effect of other covariates (such as gender) in a setting of low statistical power. As such, caution should be exercised in interpreting the finding as indicating that TEs had no effect on mental disorder.

In relation to psychosocial functioning, incremental exposure to TEs showed a strong effect at both the univariate and multivariate levels, with the largest adjusted odds ratio being observed for those who reported the highest category of TE exposure. Specifically, there was a four-fold higher prevalence of functional impairment for those with the highest exposure to past TEs even after controlling for the impact of general physical ill-health and ongoing stressors. A post-hoc multivariate test of the model adjusting for ongoing stressors (which emerged as non-significant in our univariate analyse), substantiated the relationship of high TE exposure and functional impairment. Our findings therefore are at odds with those of a previous study [41], which found that ongoing stressors, specifically denial of basic needs, significantly predicted functional impairment in displaced Darfuri refugees, an effect that was independent of past TE exposure. Contextual factors may account for these differences across studies. For example, the unresolved conflict in the homeland and lack of acknowledgment of the displaced community’s refugee status may have exacerbated the impact of past TEs on ongoing dysfunction in this group. Nevertheless, our findings caution against drawing a general inference that past TEs are of relative insignificance in predicting ongoing psychosocial functioning amongst refugees. To the contrary, our study highlights the potential enduring effects of exposure to gross human rights violations that occurred many years earlier on current psychosocial functioning.

## Conclusions

The present report is the first to document exposure to the TEs of conflict, ongoing stressors and mental health and functioning of West Papuan refugees resettled in PNG. Our findings show that the TEs of conflict, most of which occurred decades earlier in the country of origin, continued to contribute significantly to ongoing functional impairment, particularly amongst older men who had been directly involved in the resistance war in the homeland. In addition, high level of exposure to ongoing stressors was associated with current mental disorder. Given the ongoing conditions of chronic hardship and deprivation being experienced by this community, there is an urgent need for a multidimensional approach to improving the conditions in which they live, including recognizing their claims to refugee status, providing appropriate housing, security and opportunities for self-advancement, and providing accessible mental health care for those in need.
